# A fast threshold segmentation method for froth image base on the pixel distribution characteristic

**DOI:** 10.1371/journal.pone.0210411

**Published:** 2019-01-10

**Authors:** Dong-heng Xie, Ming Lu, Yong-fang Xie, Duan Liu, Xiong Li

**Affiliations:** 1 School of Information Electrical and Engineering, Hunan University of Science and Technology, Xiangtan, China; 2 School of Information Science and Engineering, Central South University, Changsha, China; 3 School of Computer Science and Engineering, Hunan University of Science and Technology, Xiangtan, China; University of Science and Technology Beijing, CHINA

## Abstract

With the increase of the camera resolution, the number of pixels contained in froth image is increased, which brings many challenges to image segmentation. Froth size and distribution are the important index in froth flotation. The segmentation of froth images is always a problem in building flotation model. In segmenting froth images, Otsu method is usually used to get a binary image for classification of froth images, this method can get a satisfactory segmentation result. However, each gray level is required to calculate each of the between-class variance, it takes a longer time in froth images with a large number of pixels. To solve this problem, an improved method is proposed in this paper. Most froth images have the pixel distribution characteristic that the gray histogram curve is a sawtooth shape. The proposed method uses polynomial to fit the curve of gray histogram and takes the characteristic of gray histogram's valley into consideration in Otsu method. Two performance comparison methods are introduced and used. Experimental comparison between Otsu method and the proposed method shows that the proposed method has a satisfactory image segmentation with a low computing time.

## 1 Introduction

In the monitoring and control of froth flotation, a key step is to analyze froth images. The size and distribution of froth are important parameters for describing flotation conditions [1,2]. Image segmentation is one of the most effective techniques for extracting those features of froth images.

In fact, there are several challenges in segmenting froth images. Froth image contains a large number of closely packed froths with weak edges affected the quality of image segmentation. A wide range of froth size and uneven illumination are other difficulties in accurate segmentation of froth images [3]. To overcome these problems, scholars developed algorithms and methods in this field. Beucher S. and Lantuejoul C. [4] developed a non-parametric method called as the watershed algorithm for contour extraction in a gray image. The watershed algorithm is an effective algorithm for segmenting froth images. However, this algorithm is easily affected by the noise, quantization error and texture details of region [5,6], so over-segmentation and under-segmentation can be found in the segmentation result. The marker-based watershed algorithm solved this weakness to some extent [7–9], and Yang et al. [10] proposed an improved watershed method based on clustering pre-segmentation and high-low scale distance reconstruction for froth images.

In order to obtain better segmentation result of froth images, Otsu method is usually used to get a binary image for marker, classification and feature extraction [11,12]. Wang WX et al. [13] used Otsu method to extract the white spot areas on froths, under which froth images are classified as the large, medium, small and mix-sized froth images, then extract the froth edges by a set of valley-edge detection algorithms based on the different gray scale distribution characteristics of each class. However, Otsu method sometimes appears under-segmentation in the segmentation of small-sized bubble images with poor illumination conditions. Gray histogram is an efficient tool in image segmentation. Some useful information or features of an image is represented by gray histogram easily [14,15]. What's more, the valley of gray histogram is a special point, most of the optimal threshold locates at the valley point or adjacent point [16,17]. Hui-Fuang Ng [18] Combined Otsu method and the valley point information, and proposed a revised Otsu method called as valley-emphasis threshold method. In valley-emphasis threshold method, the optimal threshold as close locates at the valley of the gray histogram as possible and its between-class variance is maximum. Jiu-Lun Fan and Bo Lei [19] improved Ng's method by considering the neighborhood gray information around the valley point. The valley-emphasis threshold method and its improved method can effectively segment the image. But such methods still need to calculate the data of each gray level, so the amount of calculation is large. In our work, by observing the gray histogram of a large number of froth images, We found that there is a pixel distribution characteristic in the gray histogram: the number of pixels among the adjacent gray level is quite different, so that many "peak" and "valley" appear in the gray histogram. It is appropriate to refer to the valley value for the selection of the threshold. The use of the pixel distribution characteristic can filter out useless data, which can reduce the amount of calculation.

On-line froth images processing is an important part in the process of actual flotation based on machine vision. The speed and quality of image processing affects the efficiency of the identification of the working conditions and decision-making in follow-up process control. However, the traditional Otsu method has two disadvantages in on-line froth images segmentation. Firstly, because of the large number of pixels need to be calculated, the segmentation speed is slow; Secondly, there are large differences in the number of pixels among the adjacent gray levels, it is difficult to find the best threshold. Based on the pixel distribution characteristic in the gray histogram, we combine the polynomial and the valley value to improve Otsu method, a fast threshold segmentation method is proposed in this paper. This paper is organized as follows. Firstly, the traditional Otsu method is reviewed in Section 2. Then, Section 3 presents an improved method. The result of segmentation and contrast is showed in Section 4. Finally, Section 5 concludes this paper.

## 2 Basic principle of Otsu method

Otsu method was proposed by Nobuyuki Otsu [20] and is a very famous algorithm in image segmentation field. Otsu method obtains the optimal threshold by maximizing the between-class variance functions. This method can divide the gray level of an image into two parts: foreground and background. When the difference between the two parts reaches the maximum, the corresponding threshold at this time is the optimal threshold of Otsu method. Variance is used to find the optimal threshold to segment the image, and the image after segmentation have obvious differences.

Set the image has *L* gray level, *N*_*i*_ is the number of pixels that the gray value is *i*, and the number of the total pixels is *N*.

$$N=N_{0}+N_{1}+N_{2}+\cdots +N_{L-1}$$ (1)

The probability of the pixel with gray value of *i* is *P*_*i*_ given as:

$$P_{i}=\frac{N_{i}}{N}$$ (2)

The average of gray value of an image *μ*_*T*_ is given as:

$$\mu_{T}=\sum_{i=0}^{L-1}iP_{i}$$ (3)

Let the threshold *T* divides the image into foreground *C*_0_ and background *C*_1_. Then *ω*_0_(*T*) and *ω*_1_(*T*) respectively represent the proportion of the foreground pixels and the background pixels.

$$\omega_{0}(T)=\sum_{i=0}^{T}P_{i}$$ (4)

$$\omega_{1}(T)=1-\omega_{0}(T)$$ (5)

The average of gray value of foreground *C*_0_ and background *C*_1_ is *μ*_0_ and *μ*_1_ respectively can be shown as:

$$\mu_{0}(T)=\frac{\sum_{i=0}^{T}iP_{i}}{\omega_{0}(T)}$$ (6)

$$\mu_{1}(T)=\frac{\mu_{T}-\sum_{i=0}^{T}iP_{i}}{\omega_{1}(T)}$$ (7)

The between-class variance $\sigma_{B}^{2}(T)$ is given as:

$$\sigma_{B}^{2}(T)=\omega_{0}(T){\left[\mu_{T}-\mu_{0}(T)\right]}^{2}+\omega_{1}(T){\left[\mu_{T}-\mu_{1}(T)\right]}^{2}$$ (8)

Than, select the optimal threshold T* by maximizing $\sigma_{B}^{2}(T)$:

$$\sigma_{B}^{2}(T^{*})=\underset{0\leq T\leq L-1}{\max}\sigma_{B}^{2}(T)$$ (9)

## 3 Improved method

### 3.1 Fitting function

We suppose there are N discrete points, a M-degree polynomial function *f*_*M*_(*x*,*w*) is given as [21]:

$$f_{M}(x,w)=w_{0}+w_{1}x+w_{2}x^{2}+\cdots +w_{M}x^{M}=\sum_{j=0}^{M}w_{j}x^{j}$$ (10)

Where *x*^*j*^ is the variable with an order of *j*, *w*_*j*_ is the coefficient of *j*th variable.

The fitting degree is explained by the variance between the observation of *i*th point and the value of the fitting polynomial of *i*th point, and the variance should be as small as possible. The fitting degree function *L*(*w*) is given as

$$L(w)=\frac{1}{2}\sum_{i=1}^{N}(\sum_{j=1}^{M}w_{j}x_{i}^{j}-y_{i}{)}^{2}$$ (11)

Where the coefficient $\frac{1}{2}$ is for convenience of calculation. Then,

$$\frac{\partial L(w)}{\partial w_{k}}=\sum_{i=1}^{N}(\sum_{j=0}^{M}w_{j}x_{i}^{j}-y_{i})\cdot x_{i}^{k}=0$$ (12)

By Eq (12), we can get the extreme value of fitting function.

For programming, Eq (12) is transformed into matrix operation, which is given as

$$\sum_{i=1}^{N}\sum_{j=0}^{M}w_{j}x_{i}^{j+k}=\sum_{i=1}^{N}x_{i}^{k}y_{i}$$ (13)

In actual programming, if fit with 256 data in histogram, the calculation is too large and fitting result is not good. So we divided 255 points into 5 segments, each 51 points fitted by Eq (13), and figure out the extreme value of each fitting function.

### 3.2 The segmentation algorithm

**Fig 1:**
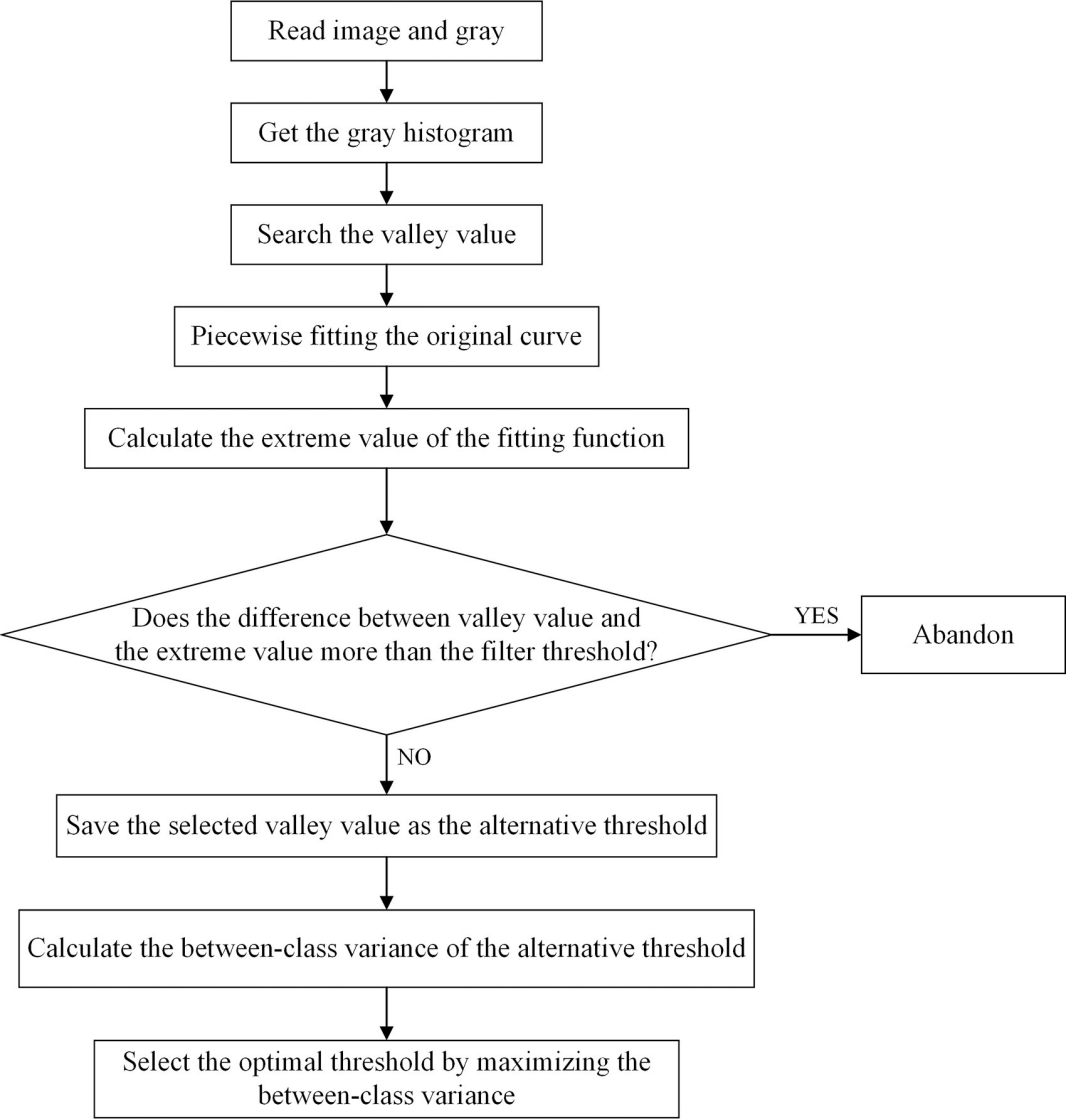
Flowchart of the improved method in this paper.

In fact, most of the optimal threshold was located in the fitting function’s valley point in the gray histogram. So we considered the extreme value, and the extreme value of the fitted curve is used as the best segmentation threshold. In Algorithm, we divide the original curve into 5 segments in this paper. That is, the number of windows is 5, and the number of gray levels for each window is 51. One parameter that needs to be set is the filter threshold. It determines the range of screening the valley near the extreme value. Then the selected valley values are saved as the alternative threshold. The filter threshold is set to 10 in this paper. The value and effect of the filter threshold are discussed in Section 4.1. The algorithm is programmed and implemented on the python platform. The flowchart of the improved method in this paper is shown in Fig 1.

Algorithm

1: Set the parameters: *T*_*F*_(the filter threshold)

2: Obtain gray value information from the read image, *G*_*h*_ is the number of pixels with a gray level of *h*

3: for *h* = 0 to 253

4:         if *G*_*h*+1_−*G*_*h*_< 0 and *G*_*h*+2_−*G*_*h*+1_>0 then

5:                 *V*_*j*_ = *G*_*h*+1_ (*V*_*j*_ is the valley value)

6:                 save *V*_*j*_ (Suppose the number of *V*_*j*_ is M)

7:         end if

8: end for

9: for i = 1 to 5 do

10:                 Select windows *W*_1_,*W*_2_,…,*W*_5_

12:                 Calculate *f*_*i*_ where is the fitting function of *W*_*i*_ according to Eq (13)

13:                 Calculate the extreme value of *f*_*i*_, and save as *x*_*k*_ (Suppose the number of *x*_*k*_ is P)

14: end for

15: for j = 1 to M do

16:         for k = 1 to P do

17:                 if |*V*_*j*_−*x*_*k*_| < *T*_*F*_ then

18:                         *T*_*r*_ = *V*_*j*_ (*T*_*r*_ is the alternative threshold)

19:                         save *T*_*r*_ (Suppose the number of *T*_*r*_ is Q)

20:                 end if

21:         end for

22: end for

23: Calculate the between-class variance of *T*_*r*_ according to Eq (8) and save as $\sigma_{B}^{2}(T_{r})$

24: Set $\sigma_{B}^{2}{\left(T_{}\right)}_{\max}$ = 0

25: for r = 1 to Q do

26:         if $\sigma_{B}^{2}(T_{r})\geq \sigma_{B}^{2}{\left(T\right)}_{\max}$ then

27:                 $\sigma_{B}^{2}{\left(T_{}\right)}_{\max}$ = $\sigma_{B}^{2}(T_{r})$

28:                 T* = *T*_*r*_

29:         end if

30: end for

## 4 Experimental results and analysis

In order to validate the proposed method in this paper, the value and effect of the filter threshold on segmentation is discussed, and we apply the proposed method and Otsu method to segment the same froth image in the experiment. In the following subsections, we present froth images and the experimental methodology. We analyze and compare the segmentation results of the proposed method and Otsu method.

In the experiment, the original Otsu method and our method are implemented in Python 3.5.2 and ran on an Intel(R) Pentium B960 Dual-Core 2.2GHz processor with a 4G RAM and a windows 10 platform. The timer starts after getting the gray histogram and ends after obtaining the optimal threshold. Then the gray-level contrast and uniformity within region of Otsu method and our method are calculated.

### 4.1 The filter threshold

The filter threshold is an important parameter in the Algorithm proposed in Section 3.2. Its value will affect the selection of the segmentation threshold and the efficiency of the algorithm. The selection criterion of the filter threshold is to ensure that the algorithm has a high correct segmentation rate and a low processing time. Therefore, the value of the filter threshold is determined by the segmentation quality and processing time of a certain number of the training images. Firstly, we set a set of the filter thresholds (5, 10, 15, 20, 25, 30). The interval of the filter thresholds is set to 5 to better highlight the differences among these filter thresholds. In the selection process, 80 froth images (20 for each class) are used to determine the filter threshold.

**Fig 2:**
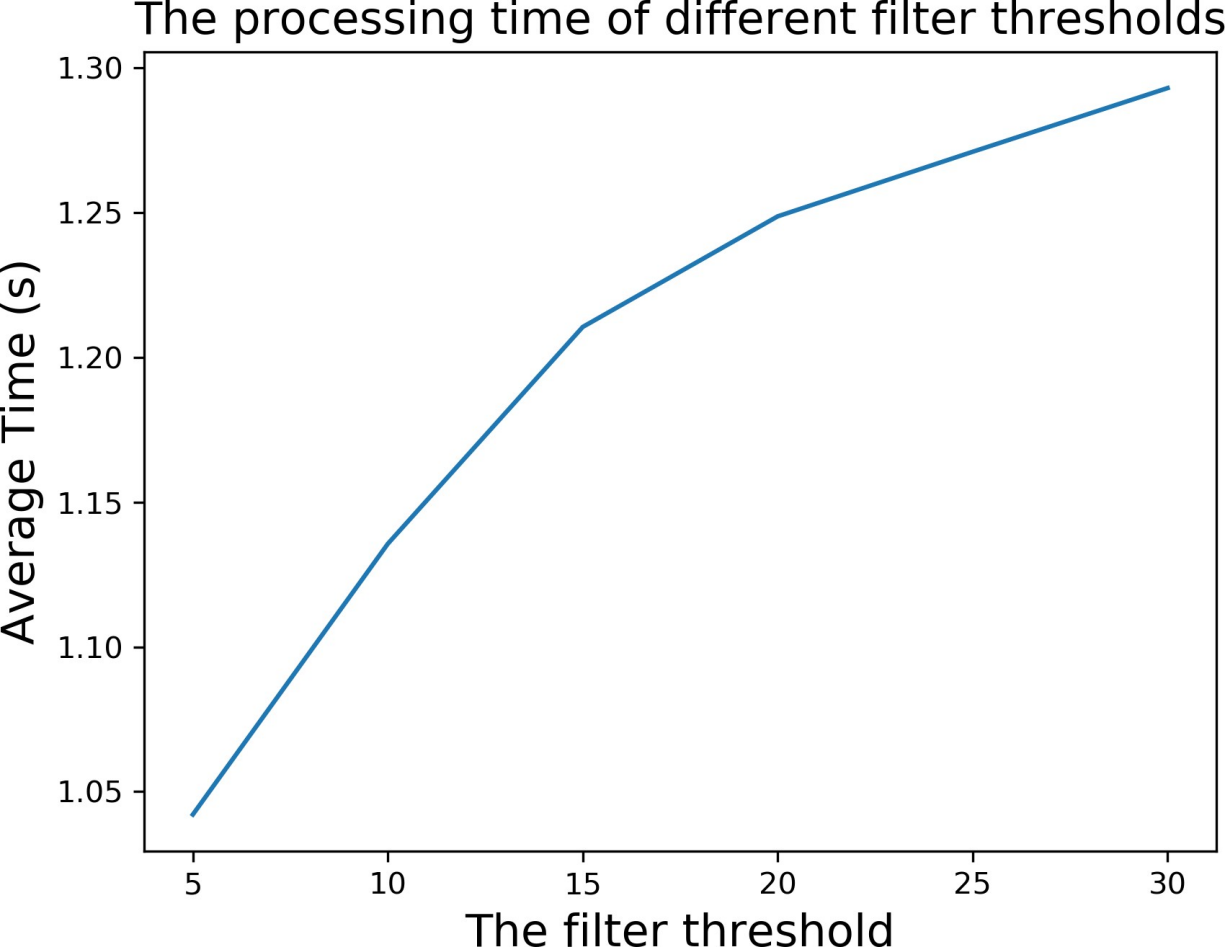
The curve of the relationship between the filter threshold and the processing time.

In terms of the processing time, as show in Fig 2, the average processing time of each image is grown with the increase of the filter threshold. This is due to the expansion of the filtering range, the number of the alternative threshold screened out by the filter threshold is increased, so the time spent on calculating and comparing the between-class variance will increase. The average time in Fig 2 is the average of the processing time of 80 times under each filter threshold.

The second consideration in selecting a filter threshold is the quality of segmentation. Based on the judgments of the human vision system, we divide the segmentation results into three categories: over-segmentation (O-S), under-segmentation (U-S), and correct-segmentation (C-S). Then the segmentation results of 80 times under each filter threshold are recorded. The statistical data are shown in the Table 1.

**Table 1:** Segmentation data of the training images.

The Filter Threshold	U-S	O-S	C-S	Accuracy rate (%)	Average Time(s)
5	5	3	72	90%	1.0421
10	2	1	77	96.25%	1.1356
15	2	1	77	96.25%	1.2106
20	6	0	74	92.5%	1.2487
25	7	0	73	91.25%	1.2710
30	7	0	73	91.25%	1.2930

In Table 1, the accuracy rate is the ratio of the number of correctly segmented images to the total number of training images. Obviously, the accuracy of the filter thresholds of 10 and 15 is higher than other filter thresholds. According to the criteria mentioned above, we choose 10 as the filter threshold.

**Fig 3:**
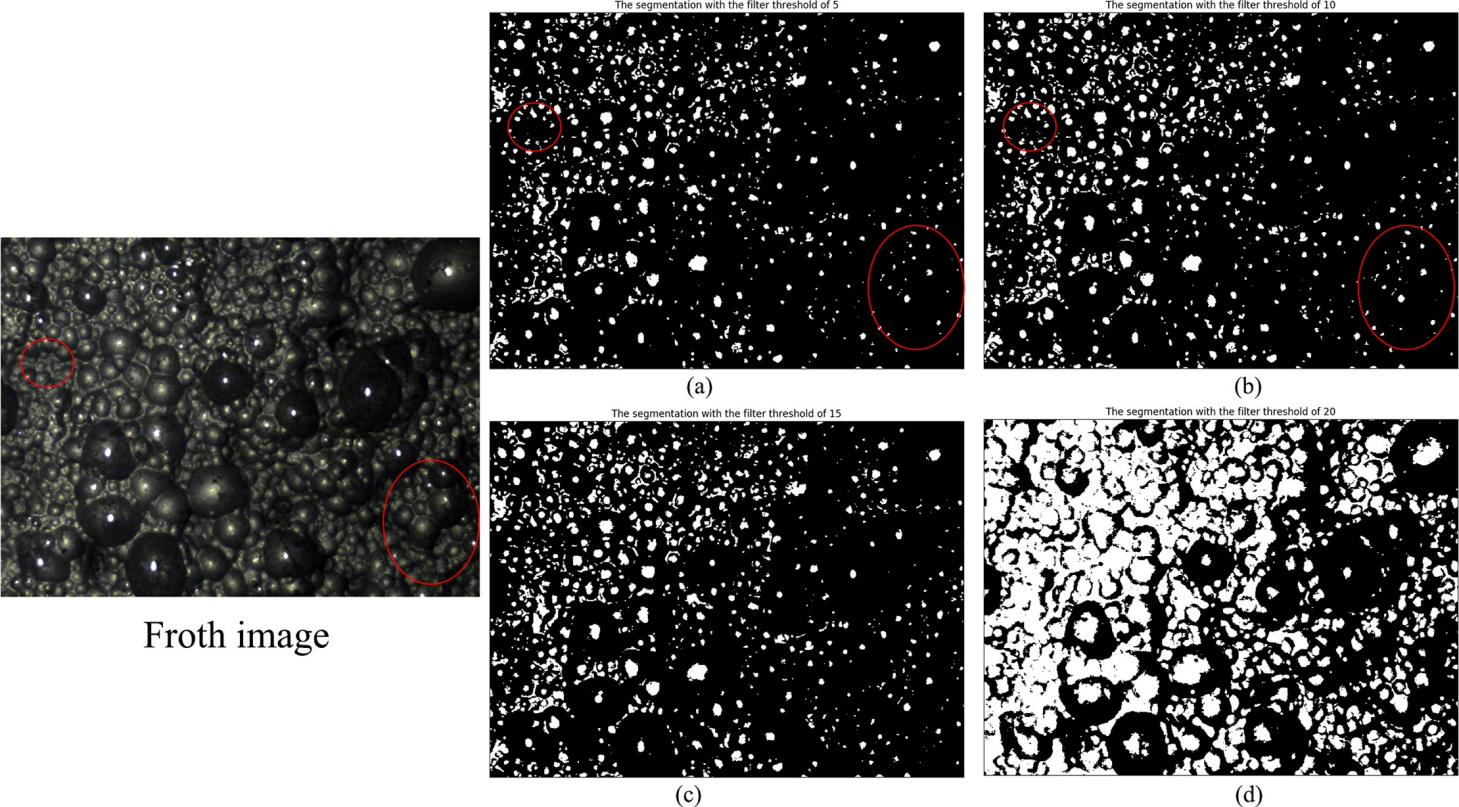
**Comparison of segmentation results with different filter thresholds in the first case.** (a, b, c and d are the segmentation result with the filter thresholds of 5, 10, 15 and 20, respectively).

**Fig 4:**
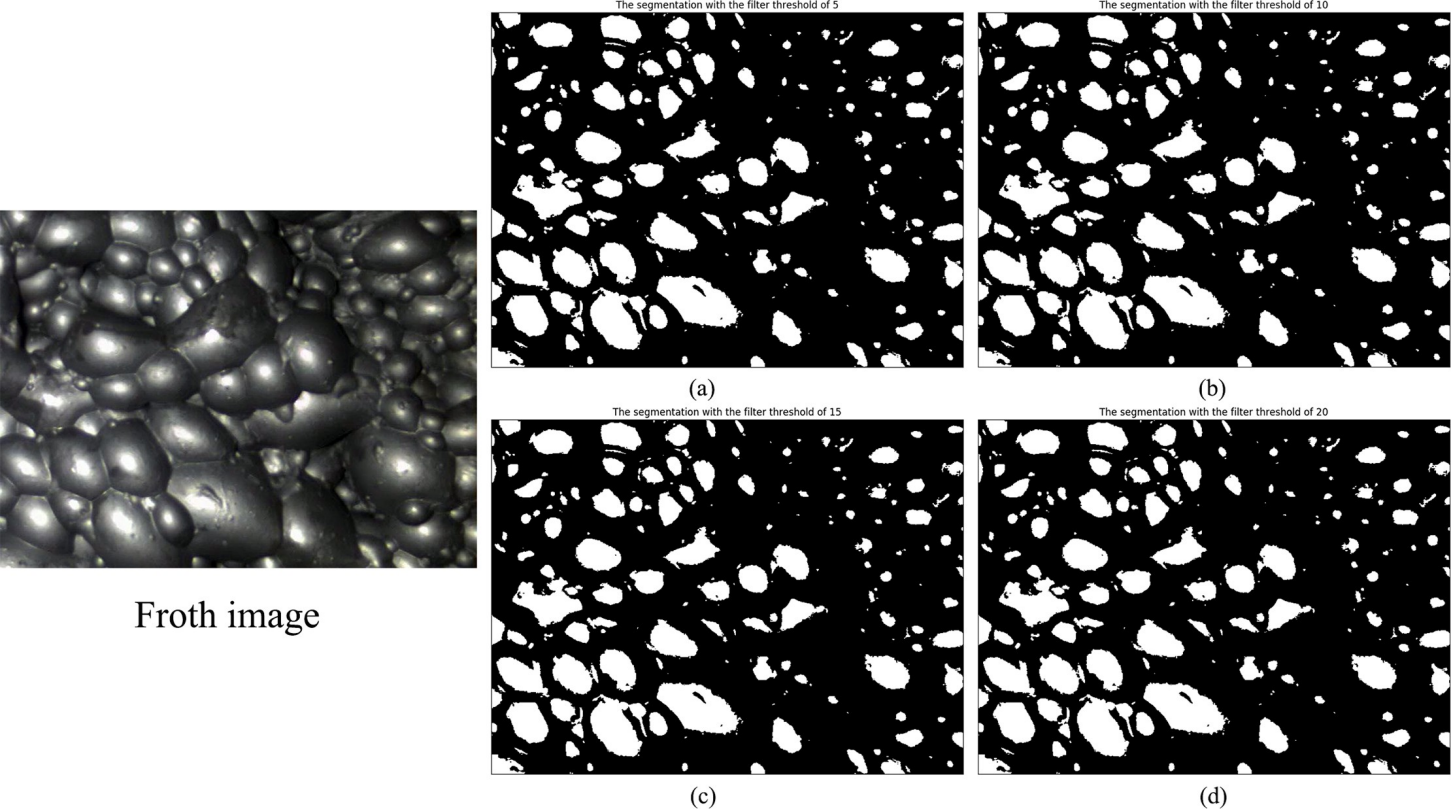
**Comparison of segmentation results with different filter thresholds in the second case.** (a, b, c and d are the segmentation result with the filter thresholds of 5, 10, 15 and 20, respectively).

Next, we will discuss the impact of the filter threshold on the algorithm in two detailed cases. The segmentation results under each the filter threshold are shown in Fig 3 and Fig 4, and the detailed comparison data are given in Table 2.

**Table 2:** The comparison of data under different filter thresholds.

Froth image	The filter threshold	Threshold	The number of the valley	The number of the alternative threshold	Average time(s)
**The first case**	5	96	75	29	1.0434
	10	91		43	1.1302
	15	87		53	1.2010
	20	45		62	1.2502
**The second case**	5	103	72	21	1.0102
	10	103		39	1.0899
	15	103		53	1.1862
	20	103		61	1.2548

From the above experimental results, the inappropriate filter threshold will affect the segmentation quality and processing time of the algorithm. More segmentation results of froth images are discussed in the next section.

Firstly, in the quality of segmentation, the segmentation with the filter threshold of 5 appears over-segmentation in some places. Part of small bubbles is lost, which is marked in the Fig 3. The segmentation results with the filter threshold of 10 and 15 are relatively good. However, there is a clear under-segmentation in the segmentation results when the filter threshold is 20. Because of the enlargement of the filtering range, some useless values are filtered as the alternative thresholds. Sometimes the between-class variance of these values is the largest among all the alternative thresholds. This causes the algorithm to select a poor threshold, resulting in under-segmentation in the segmentation result. Therefore, the filter threshold should not be too large. Otherwise, it can’t guarantee the validity of the extreme value very well. In Fig 4, the segmentation results are consistent under different filter thresholds. Due to the between-class variance of the threshold 103 is the largest of all gray levels and one extreme value of the fitting function is 100, the optimal threshold selected by the proposed method is always 103 when the filter threshold is increased.

Secondly, the processing speed of the algorithm is related to the filter threshold. Table 2 shows that as the filter threshold increases, the number of the alternative threshold also increases. As a result, the algorithm needs to calculate and compare the between-class variance of more alternative thresholds. Correspondingly, the processing time also increases.

### 4.2 Comparison of segmentation between the proposed method and Otsu

**Fig 5:**
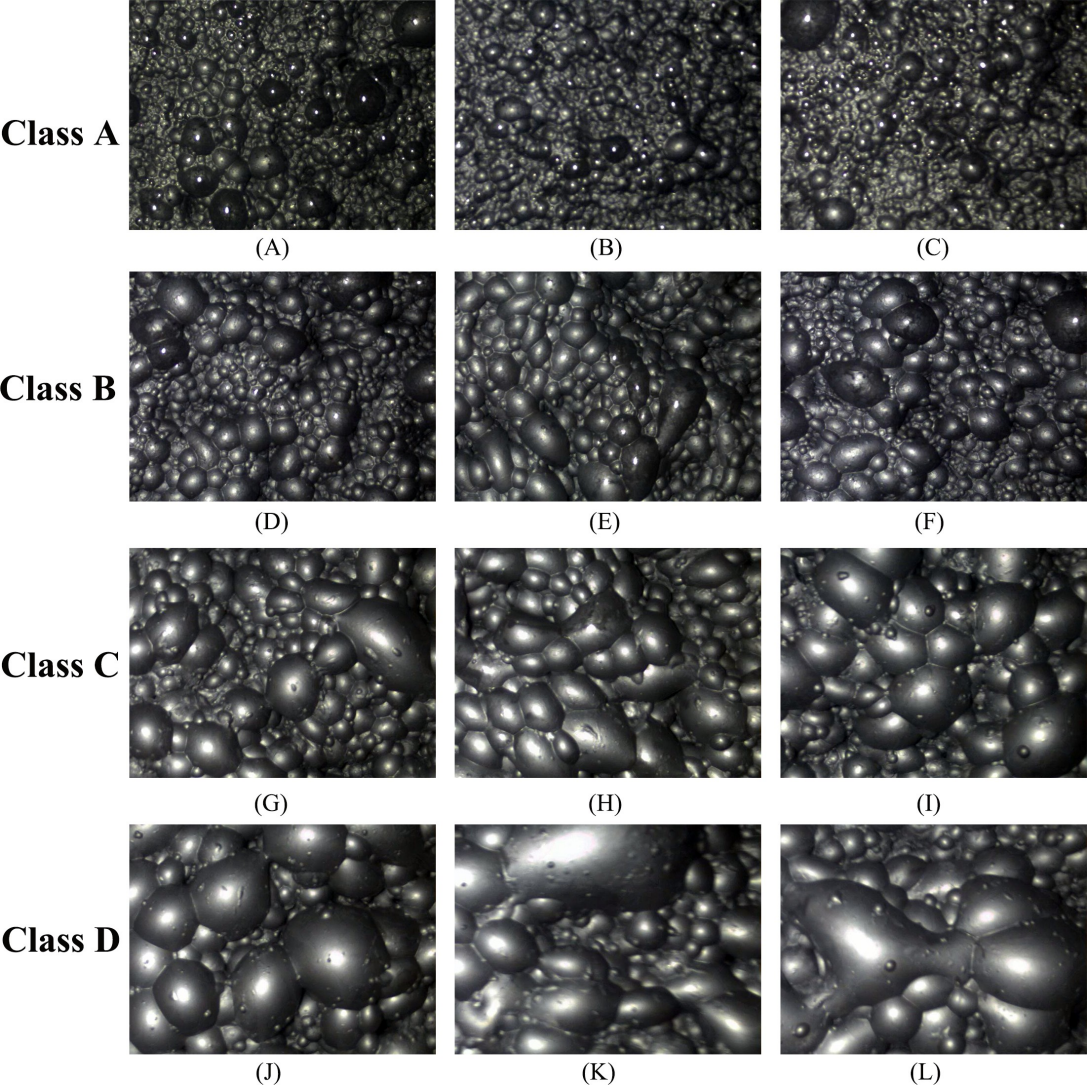
Origin image.

**Fig 6:**
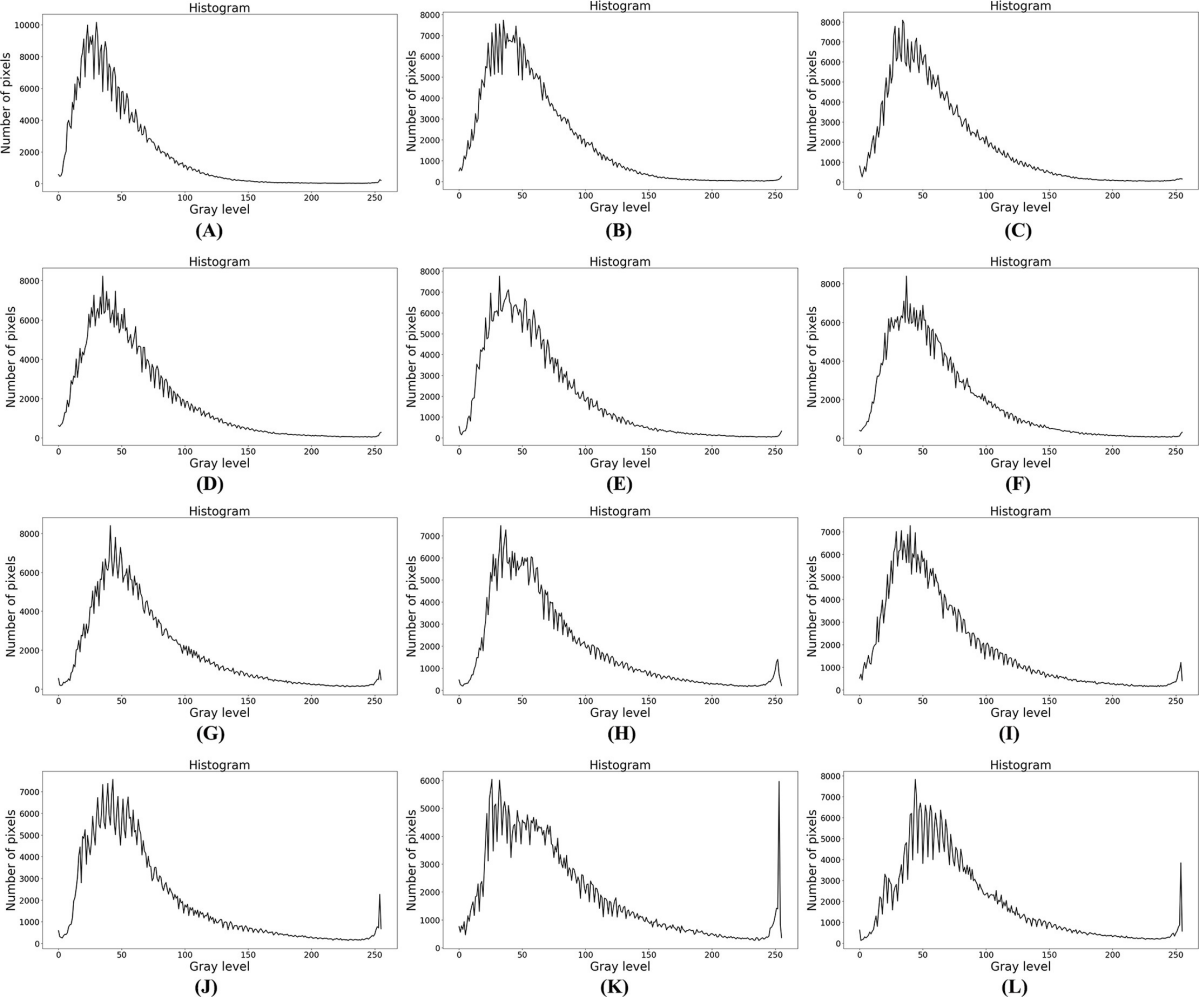
Gray histogram.

According to the four types of froth listed in the literature [3], four classes of froth images are selected, and a total of 12 images are used in the experiment to test the performance of the proposed method and Otsu method. The size of each image is 800×600. The froth in class A images are mostly very small spherical bubbles with a very large number. Most froth in class B are small in size, irregular in shape and large in number. The class C images contain froths of medium size, good structure and medium number. The size of the froth in the class D image is large and the number of froth is small. 12 froth images and their gray histogram are shown in Figs 5 and 6 respectively. In Fig 6, the shape of each curve in gray histogram is sawtooth, and there are many peaks and valleys on the whole curve.

**Fig 7:**
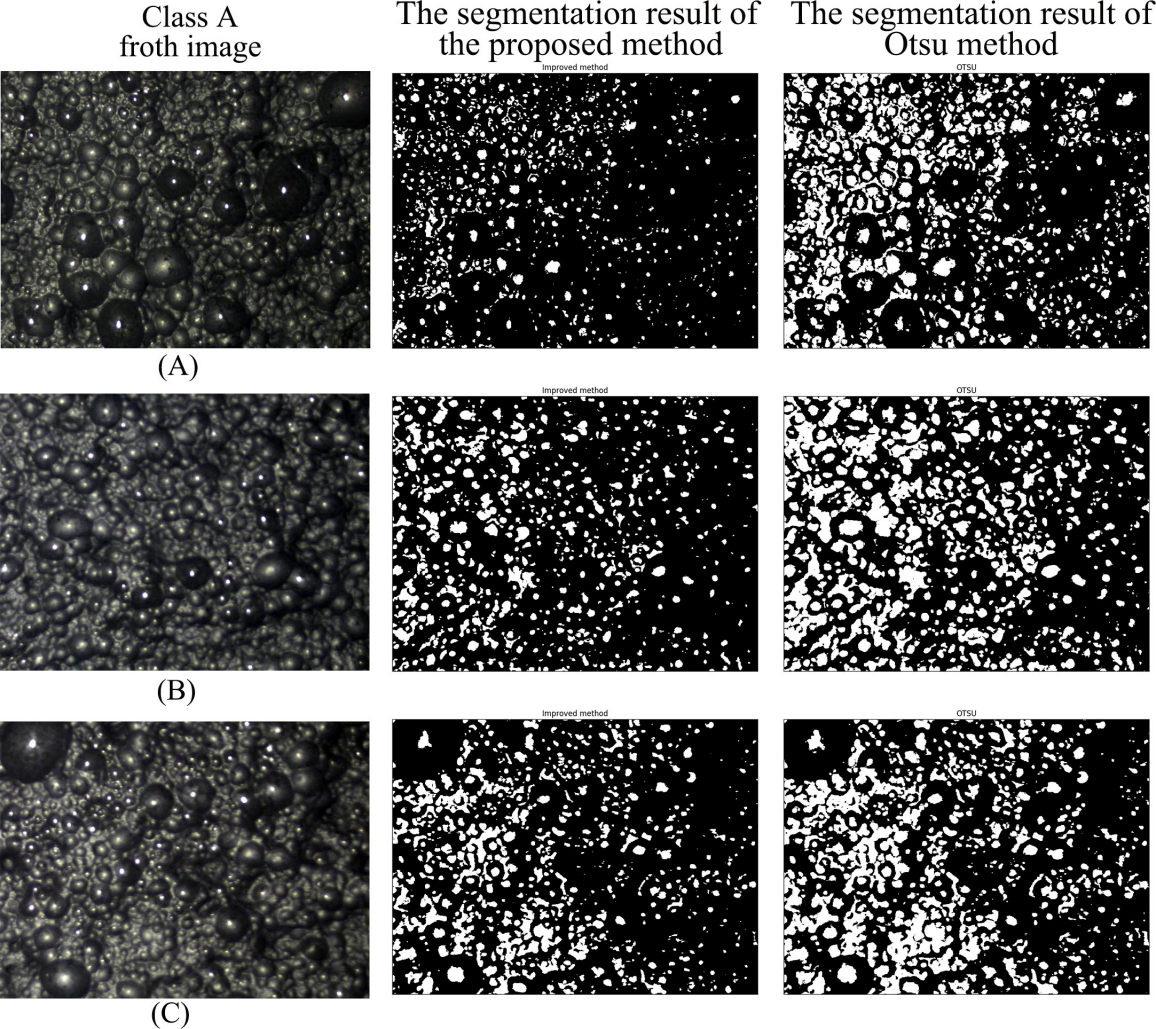
The segmentation result of class A froth image.

**Fig 8:**
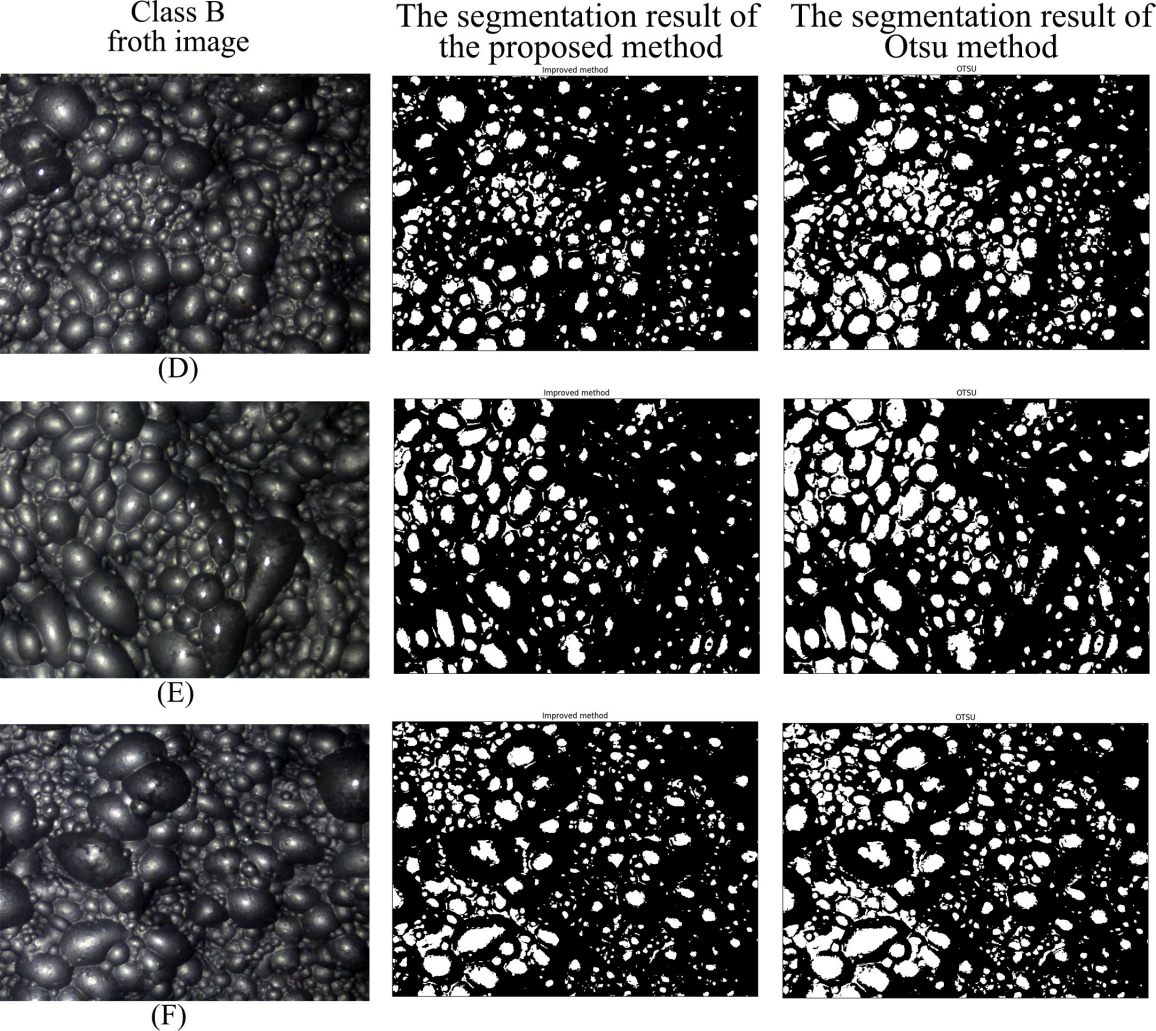
The segmentation result of class B froth image.

**Fig 9:**
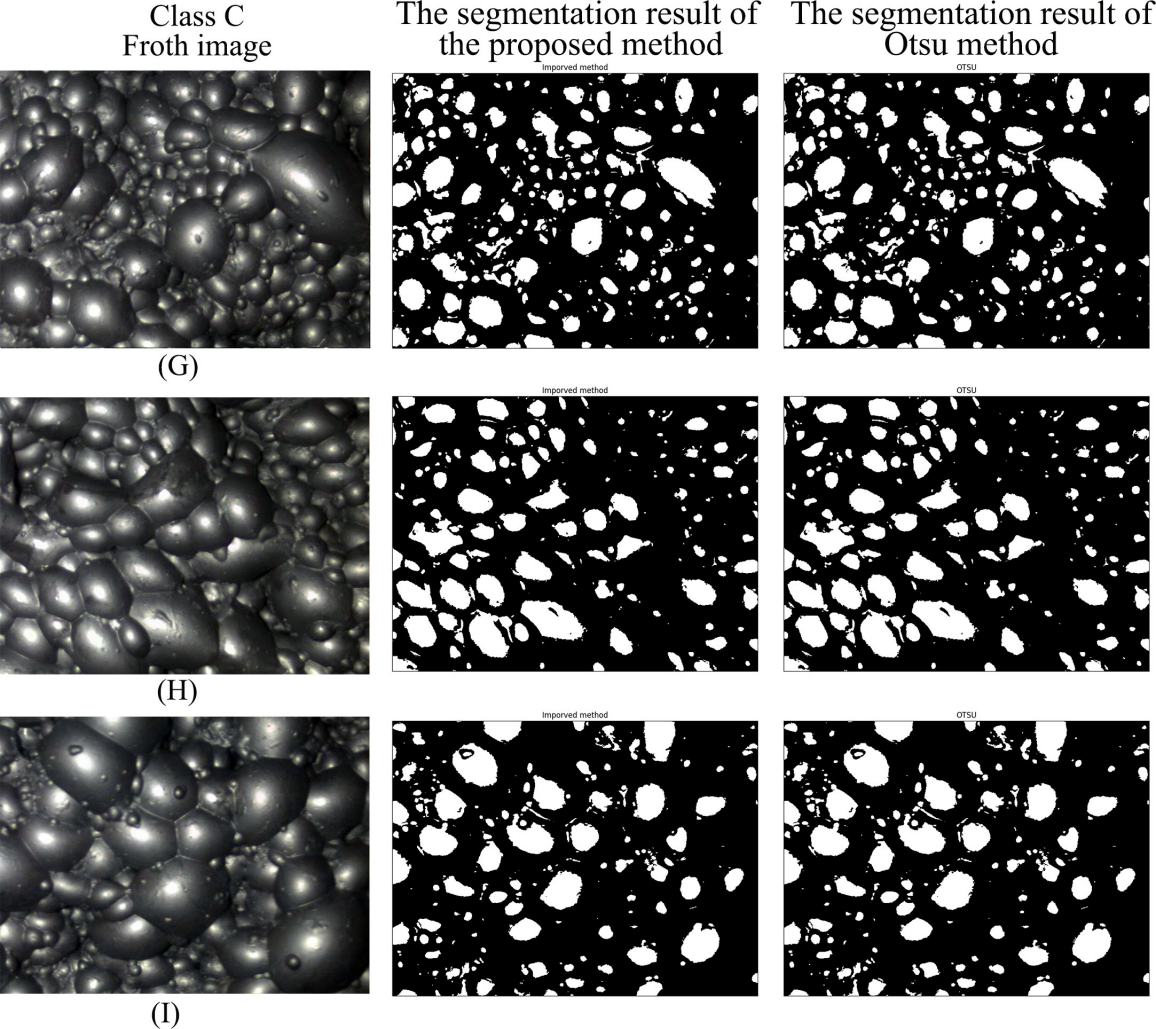
The segmentation result of class C froth image.

**Fig 10:**
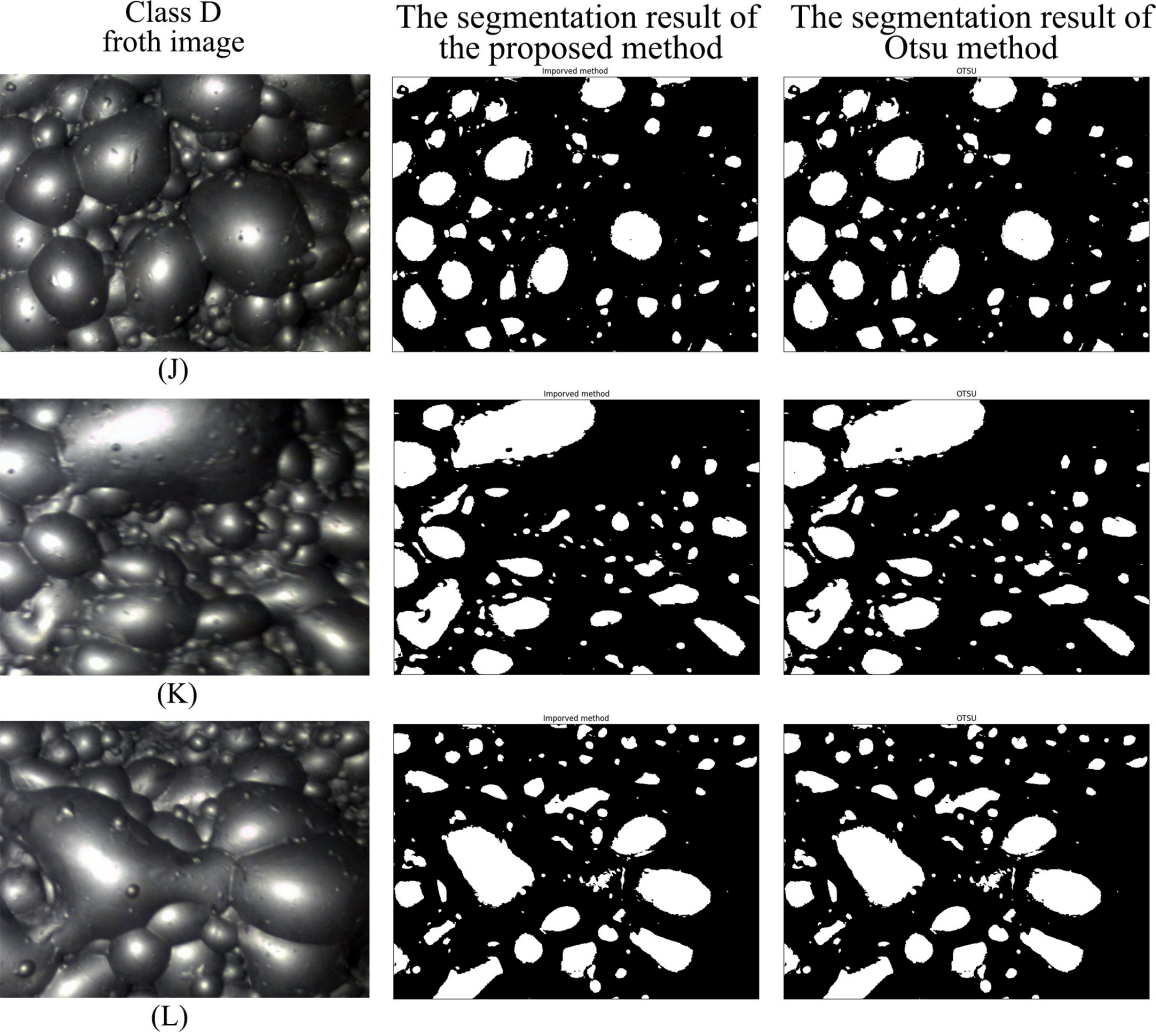
The segmentation result of class D froth image.

Figs 7–10 are the segmentation results of the proposed method and Otsu method on the froth images of class A, class B, class C and class D, respectively. In Figs 7 and 8, the segmentation result of Otsu method shows an obvious under-segmentation. Some small froths were not effectively segmented. However, after combining the pixel distribution characteristics with Otsu method, the proposed method can effectively segment the small bubbles. It performs well in the segmentation results of class A and class B.

Figs 9 and 10 show that both the proposed method and Otsu method can effectively segment class C and class D froth images. Due to the threshold obtained by the two methods are similar or identical, the two methods are close in the segmentation result. But the average processing time of the proposed method is much shorter than Otsu method. Detailed comparison data are presented in Table 3.

**Table 3:** Comparative data.

Images number	The proposed method (Otsu method)			
	Threshold	Average Time(s)	UM	GC
**A**	91 (62)	1.1302 (1.6543)	0.5189 (0.6165)	0.5112 (0.4773)
**B**	90 (70)	1.1880 (1.6351)	0.5809 (0.6315)	0.4470 (0.4391)
**C**	91 (77)	1.1921 (1.6423)	0.6218 (0.6419)	0.4534 (0.4479)
**D**	92 (77)	1.1472 (1.6488)	0.6189 (0.6400)	0.4725 (0.4641)
**E**	92 (80)	1.0006 (1.6279)	0.6236 (0.6368)	0.4610 (0.4525)
**F** **G** **H** **I** **J** **K** **L**	92 (81) 97 (97) 103 (103) 98 (97) 104 (102) 113 (114) 110 (110)	1.1292 (1.6244) 1.0746 (1.6411) 1.0899 (1.6593) 1.2129 (1.6397) 1.0954 (1.6220) 1.2064 (1.6324) 1.0310 (1.6402)	0.6338 (0.6447) 0.6607 (0.6607) 0.6759 (0.6759) 0.6627 (0.6627) 0.6747 (0.6749) 0.6984 (0.6984) 0.6640 (0.6640)	0.4675 (0.4617) 0.4664 (0.4664) 0.4882 (0.4882) 0.5038 (0.5032) 0.5105 (0.5092) 0.5078 (0.5079) 0.4601 (0.4601)

**Fig 11:**
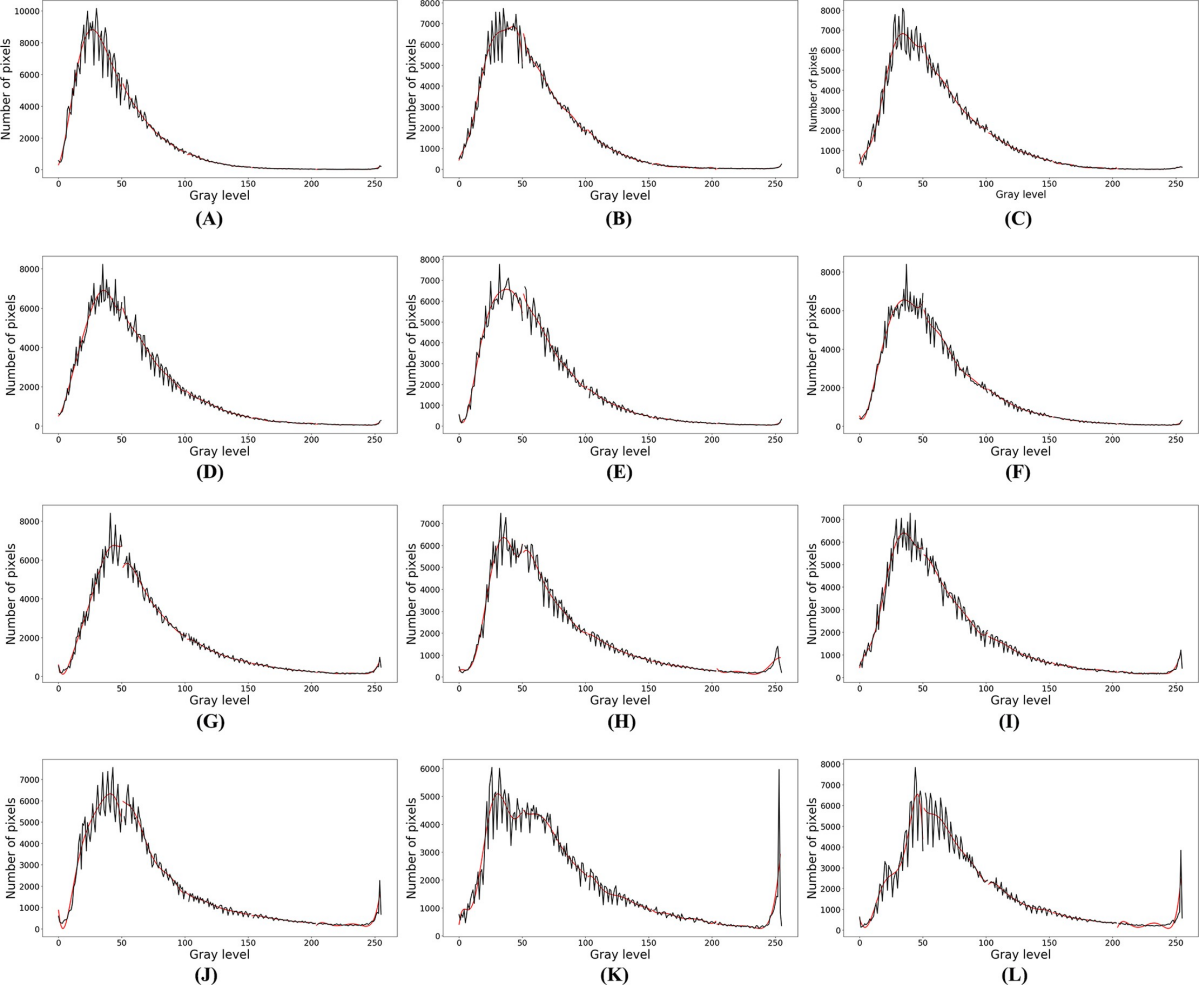
The original curve and the fitting curve.

In the process of fitting the original curve, the setting of polynomial order is a major problem. If the order is too low or too high, it may lead to under-fitting or over-fitting. In order to obtain the best between the fitting precision and the complexity of the fitting function for the froth images in the experiment, we set the order to 6. The original curve of gray histogram is divided into 5 parts, each part is fitted by the method proposed in Section 3.1. It can fit the original curve well, and the complexity is low, as well as the algorithm can be ran quickly. The original curve and the fitting curve of 6 froth images are shown in Fig 11, in which the dark line and the red line are the original curve and the fitting curve respectively.

### 4.3 Gray-level contrast and Uniformity within region

Gray-level contrast

In literature [22], suppose the segmented image has two regions, one of regions is *f*_*o*_, and the other is *f*_*b*_. The gray-level contrast of the two regions GC is given as

$$GC=\frac{\left|f_{o}-f_{b}\right|}{f_{o}+f_{b}}$$ (14)

GC is seen to have a minimum value of zero and a maximum value of one. For the segmented froth image in this paper, the value of GC of the segmented images should be greater than 0.44.

2) Uniformity within region

In [22], set a given threshold value t, and the uniformity measure UM is given as:

$$UM=1-\frac{\sigma_{1}^{2}+\sigma_{2}^{2}}{C}$$ (15)

Where

$$\sigma_{i}^{2}=\sum_{(x,y)\in R_{i}}{\left(f(x,y)-\mu_{i}\right)}^{2}$$ (16)

$$\mu_{i}=\frac{\sum_{R_{i}}f(x,y)}{A_{i}}$$ (17)

*R*_*i*_ is the *i*th segmentation region, *f*(*x*,*y*) is the gray level of the pixel (*x*,*y*), the number of pixels in *R*_*i*_ is *A*_*i*_, and *C* is a normalization factor. The range of UM value is zero to one. For the segmented froth image in this paper, the UM value of the segmented image should be greater than 0.5.

In order to obtain accurate data for comparison, the segmentation of each image is repeated 10 times. The processing time is the average value among the 10 groups of data. The optimal threshold, GC, UM and the processing time of the two methods are recorded and compared in Table 2. Both methods of GC and UM have reached the standard. The GC of the proposed method is generally slightly larger than Otsu method, and it also shows that the segmentation result of the proposed method is more distinguishing than Otsu method. Otsu method calculates the between-class variance of all gray levels and selects the gray level of the maximum between-class variance. The segmented image has good uniformity, so its UM is slightly greater than the proposed method. In the term of processing time, the average processing time of Otsu method is 1.6220s-1.6593s, and the average processing time of the proposed method is 1.0006s-1.2064s. Obviously, the proposed method is faster than Otsu method in processing speed.

### 4.4 Experiment analysis

The speed and quality of segmentation are two key points in on-line froth images segmentation. It can be concluded from the previous experiments that the proposed method can effectively segment 4 classes of froth images, and is superior to the Otsu method in the segmentation of class A and class B froth images. The segmentation results obtained by the proposed method are generally highly distinguishable. Moreover, the proposed method is faster than Otsu method in the processing speed.

In addition, the value of the filter threshold is a key point in the proposed method. If it is too low, the number of reference points is small, and the threshold selected by the proposed method is not necessarily optimal. When the filter threshold is too high, the number of alternative thresholds is large, so that the number of useless points is increased. It not only increases the amount of calculation but also affects the selection of the optimal threshold. Therefore, the selection of the filter threshold is very important. A suitable filter threshold enables the algorithm to have satisfactory accuracy and reduce the calculation of useless points.

## 5 Conclusions

According to different processing objects, it is necessary to get better segmentation results by selecting or optimizing segmentation methods. In the process of on-line froth image segmentation, it not only requires fast segmentation speed but also ensures the quality of the segmentation. Therefore, we studied and combined the distribution characteristics of the pixels in froth images to improve Otsu method for froth images segmentation.

In this paper, we present a fast threshold segmentation method for froth image base on the pixel distribution characteristic. First, a piecewise fitting is used to fit the curves in the histogram. On the basis of the obtained extreme values, the filter threshold is set to filter the valley value of the gray histogram as the alternative thresholds. Then, from all the alternative thresholds, the one with the largest between-class variance is selected as the optimal threshold. As shown in the experiment, the proposed method in this paper can segment froth images in a fast and effective way which has an obvious advantage over Otsu method.
